# Non-Surgical Management of Gallstones During Pregnancy: A Clinical Case Report

**DOI:** 10.7759/cureus.76560

**Published:** 2024-12-29

**Authors:** Adrian Boicean, Liana Chicea, Victor Tudor, Radu Chicea, Flavia Tudor, Romeo-Gabriel Mihaila, Cosmin Nicodim Cindea

**Affiliations:** 1 Gastroenterology, County Clinical Emergency Hospital of Sibiu, Sibiu, ROU; 2 Medicine, Lucian Blaga University of Sibiu, Sibiu, ROU; 3 Internal Medicine, County Clinical Emergency Hospital of Sibiu, Sibiu, ROU; 4 Obstetrics and Gynecology, Lucian Blaga University of Sibiu, Sibiu, ROU; 5 Hematology, Sibiu County Emergency Clinical Hospital, Sibiu, ROU; 6 Neurosurgery, County Clinical Emergency Hospital of Sibiu, Sibiu, ROU; 7 Surgery, Lucian Blaga University of Sibiu, Sibiu, ROU

**Keywords:** bile duct stent, cholelithiasis, gallstones, hormonal factors, maternal outcomes, omega-3 fatty acids, pregnancy, ursodeoxycholic acid

## Abstract

Gallstone disease during pregnancy, or cholelithiasis, presents significant clinical challenges due to hormonal, anatomical, and metabolic changes. Progesterone therapy, commonly used in pregnancy for uterine bleeding, can exacerbate gallstone risk by reducing gallbladder motility and promoting cholesterol gallstone formation. This case report describes a 29-year-old pregnant woman with no prior gallbladder disease who developed multiple cholesterol gallstones during the third trimester while undergoing progesterone therapy for bleeding associated with a bicornuate uterus. Conservative management during pregnancy, including dietary modifications and close monitoring, was successful, and the patient delivered a healthy infant via cesarean section.

Postpartum, the patient developed obstructive jaundice, severe right hypochondriac pain, and scleral icterus due to common bile duct obstruction from gallstones. Endoscopic retrograde cholangiopancreatography (ERCP) with biliary stent placement resolved the obstruction, and pharmacological treatment with ursodeoxycholic acid (UDCA) and omega-3 fatty acids led to complete gallstone resolution within three months. Surgical intervention was avoided to prioritize postpartum recovery and breastfeeding, which resumed successfully after a brief interruption.

This case highlights the value of individualized, multidisciplinary care in managing pregnancy-associated gallstone disease. Conservative approaches, including pharmacological and minimally invasive interventions, can achieve effective outcomes while minimizing maternal-fetal risks. Routine ultrasound screening in high-risk pregnancies and further investigation into UDCA and omega-3 therapies, progesterone-related gallbladder stasis, and postpartum biliary stenting protocols are recommended to optimize management strategies.

## Introduction

Pregnancy is a dynamic physiological state involving metabolic and hormonal changes, including a heightened risk of gallstone formation (cholelithiasis), which can affect maternal and fetal health. Elevated estrogen and progesterone levels alter bile composition and gallbladder motility: estrogen promotes hepatic cholesterol secretion, increasing bile saturation, while progesterone reduces gallbladder contractility, causing bile stasis. These factors, combined with increased cholesterol secretion and reduced motility, significantly contribute to gallstone formation, especially in the third trimester [1].

Approximately 5% of pregnant women develop gallbladder sludge or gallstones, with 1.2% experiencing biliary pain. Most cases are asymptomatic and managed conservatively through dietary changes, pharmacological treatments, and monitoring. However, untreated symptomatic cases may lead to complications such as biliary obstruction and pancreatitis, requiring careful management, ranging from conservative approaches to surgical intervention [2].

Gallstones, solid deposits in the gallbladder, vary in composition and consistency, influencing treatment efficacy. Cholesterol gallstones, comprising 80% of cases, are softer and more responsive to therapies like chenodeoxycholic acid (CDCA), which reduces bile cholesterol saturation. CDCA achieves up to 60% dissolution success under optimal conditions but may require prolonged treatment (up to two years) and can cause side effects like diarrhea and liver enzyme abnormalities. Harder, bilirubin-based pigment stones are less responsive to dissolution therapies [3].

Omega-3 polyunsaturated fatty acids (PUFAs), such as eicosapentaenoic acid (EPA) and docosahexaenoic acid (DHA), have shown potential in gallstone management when combined with ursodeoxycholic acid (UDCA). Animal studies suggest PUFAs reduce cholesterol crystal formation by suppressing mucin secretion and may help prevent recurrence by modulating bile composition. However, their role in clinical practice remains under investigation [4].

Cholecystectomy, the gold standard for symptomatic gallstones, has high success rates but carries risks. Laparoscopic cholecystectomy has a mortality rate of 0.1-0.3% and morbidity of 2-6%, with complications including bile duct injuries (0.3-0.5%), bile leaks (0.3-0.6%), and postcholecystectomy syndrome (PCS) in 5-40% of cases. Open cholecystectomy has higher mortality rates (0.5-1%) in emergencies or patients with comorbidities [5-6]. These risks highlight the importance of individualized care and comprehensive follow-up.

Gallstone disease is one of the most prevalent gastrointestinal conditions during pregnancy, caused by hormonal and physiological changes that promote bile stasis and lithogenic bile. While surgery is well-established, conservative management is often the first line, particularly in the first and third trimesters when surgical risks to the mother and fetus are elevated. Treatments include dietary modifications, pharmacotherapy, and close monitoring, though guidelines remain limited due to insufficient data on efficacy and safety [7].

This article presents a unique postpartum case of gallstone resolution achieved entirely through minimally invasive techniques and pharmacological management, avoiding surgical intervention. It emphasizes the significance of tailoring treatment to individual postpartum challenges, such as breastfeeding and recovery, while advocating for the integration of routine gallstone screening in high-risk pregnancies.

## Case presentation

Initial diagnosis during pregnancy

**Figure 1:**
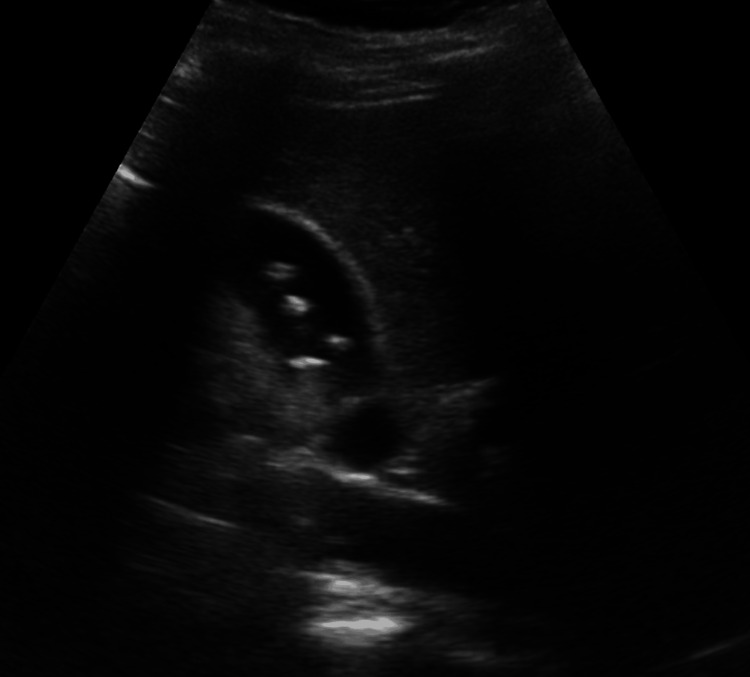
Ultrasound: multiple cholesterol gallstones (4-9 mm in size) Multiple cholesterol gallstones are observed at the level of the gallbladder without a posterior acoustic shadow. Five stones are visible in this section, with more than 10 in total.

A 29-year-old pregnant woman, with no prior history of gallbladder disease, was diagnosed with multiple gallstones during her third trimester. Ultrasound imaging (Figure 1) revealed multiple cholesterol gallstones measuring 4-9 mm in size. Notably, the patient had been undergoing high-dose progesterone therapy throughout her pregnancy to manage uterine bleeding caused by a bicornuate uterus, a medical treatment that likely exacerbated gallstone formation by reducing gallbladder motility [8]. Despite these findings, the patient remained asymptomatic and, in consultation with her care team, opted for a conservative “wait-and-see” approach, which was an accepted strategy in the absence of significant symptoms. She underwent cesarean delivery due to obstetric indications, resulting in the birth of a healthy 3.6 kg baby with a 10/10 APGAR (appearance, pulse, grimace, activity, and respiration) score.

Postpartum presentation

**Figure 2:**
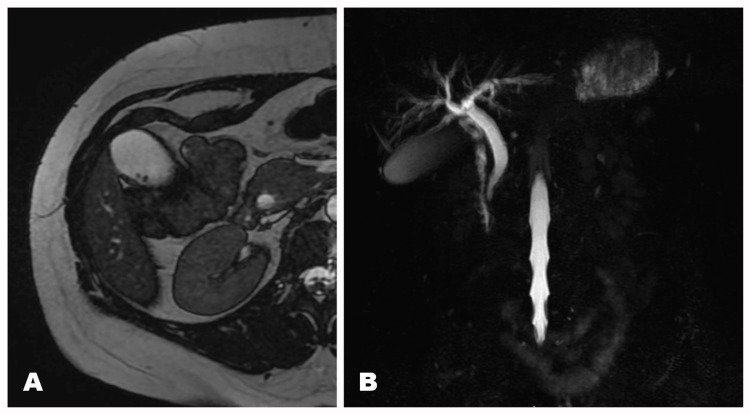
Abdominal MRI - obstructive jaundice of the common bile duct with multiple cholesterol gallbladder stones (A) T2-weighted axial MRI showing multiple infracentimetric gallstones within the gallbladder; (B) magnetic resonance cholangiopancreatography (MRCP) revealing distal segment obstruction of the common bile duct.

Two weeks postpartum, the patient presented with severe right hypochondriac pain, jaundice, and yellow sclera. Laboratory findings indicated obstructive jaundice, and imaging studies, including MRI and ultrasound, confirmed multiple gallstones with one stone obstructing the common bile duct (Figure 2).

Therapeutic decision and endoscopic intervention

Having recently given birth, the patient faced significant emotional and physical challenges of early motherhood. She expressed concerns regarding surgical intervention, including potential disruption to breastfeeding, separation from her newborn, and surgical risks that could impact her maternal role during the critical postpartum bonding period.

**Figure 3:**
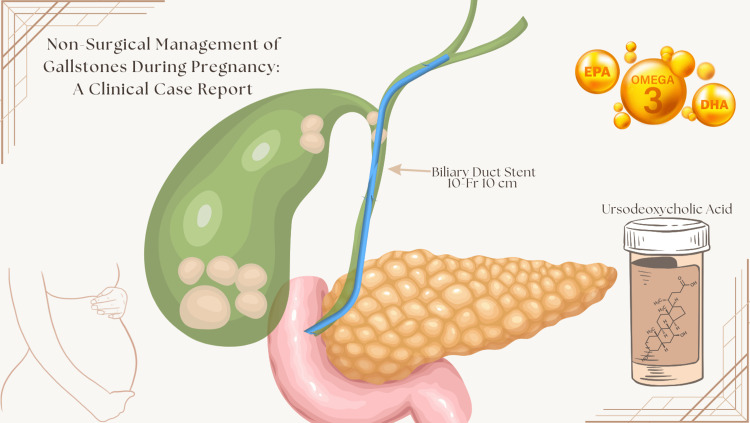
Graphical representation of the 10-French stent placement and multiple cholesterol gallstones Image credits: Cosmin Cindea. Created using Canva Pro under an active subscription.

Given these concerns, a conservative, minimally invasive treatment strategy was pursued. All endoscopic interventions were performed within a one-day surgery framework to minimize disruption to her routine. Procedures included upper gastrointestinal endoscopy for diagnostic confirmation. Endoscopic retrograde cholangiopancreatography (ERCP) for bile duct clearance and biliary stent placement (10-French, 10 cm). Subsequent biliary stent removal following symptom resolution (Figure 3).

Lactation was interrupted for only 24 hours post-ERCP due to the use of propofol anesthesia, aligning with guidelines for breastfeeding safety post-anesthesia.

Pharmacological management, resolution, and follow-up

Post-ERCP, the patient was started on a combined pharmacological regimen consisting of UDCA at 25 mg/kg/day to promote bile solubility and omega-3 fatty acid supplementation (620 mg EPA, 410 mg DHA per day) to reduce cholesterol saturation in bile.

**Figure 4:**
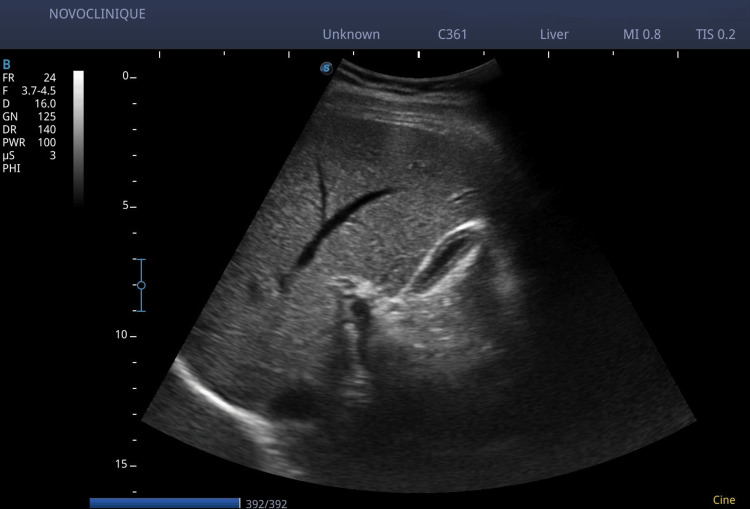
Biliary ducts ultrasound at three-month follow-up: complete resolution of the gallstones The postprandial gallbladder is visualized and emptied of bile, with no gallstones detected. There are no signs of pathological changes observed in the gallbladder or bile ducts.

Weekly ultrasound monitoring demonstrated a gradual reduction in gallstone size and number. By the third week following treatment initiation, only two small stones remained, with the largest measuring 7 mm in diameter. At eight weeks, follow-up imaging confirmed a complete resolution of gallstones (Figure 4).

The stent was removed after eight weeks. Over the following three years, the patient remained asymptomatic, with no recurrence of gallstones. Prophylactic doses of UDCA and omega-3 supplementation were continued for six months postpartum to maintain bile stability and prevent recurrence.

This case underscores the efficacy of a conservative, minimally invasive approach to managing pregnancy-associated gallstones complicated by obstructive jaundice. Key components of successful management included the following:

- Endoscopic intervention: ERCP offered an effective and safe solution for bile duct obstruction, avoiding the need for surgical cholecystectomy in the immediate postpartum period.

- Pharmacological therapy: UDCA and omega-3 fatty acids contributed to gallstone dissolution and prevention of recurrence by improving bile composition.

- Patient-centered care: Addressing the patient’s concerns about breastfeeding, surgical risks, and maternal bonding was integral to the chosen treatment strategy.

Video 1 captures the ultrasonographic evolution of the condition presented in this article.

**Video 1:** Multiple cholesterol gallstones in pregnancy - stent implantation - medical treatment - resolution

## Discussion

This case illustrates the significant impact of hormonal and structural factors on gallstone formation during pregnancy. The patient’s high-dose progesterone treatment for uterine bleeding due to a bicornuate uterus likely contributed to gallbladder stasis and cholesterol gallstone development. The asymptomatic course during pregnancy permitted a conservative “wait-and-see” approach, underscoring the importance of individualized care in such cases [9]. Additional factors such as maternal age, obesity, and ethnicity can further exacerbate the risk of gallstone formation during pregnancy. These hormonal changes disrupt normal bile flow, facilitating the formation of gallstones, particularly in women with other risk factors.

The postpartum progression to obstructive jaundice highlights the unpredictable nature of gallstone disease in this population. Timely ERCP with stent placement, paired with pharmacological management using UDCA and omega-3 supplementation, offered a minimally invasive solution. UDCA enhances bile flow, reduces cholesterol saturation, and improves the solubility of bile acids, helping to promote the dissolution of cholesterol stones. Omega-3 fatty acids, such as EPA and DHA, improve bile composition by reducing mucin production and maintaining a favorable cholesterol nucleation time. This strategy not only resolved symptoms but also minimized the disruption to the patient’s role as a new mother. Patient-centered care was crucial in this case. Addressing the mother’s concerns about breastfeeding and postpartum recovery shaped the decision for non-surgical management. This underscores the need for multidisciplinary approaches that balance maternal and neonatal well-being with effective treatment [10-11]. 

Temporary biliary stents have proven useful in managing acute biliary obstruction during pregnancy. Stents can provide immediate relief by bypassing the obstruction, preventing further complications like cholangitis or pancreatitis. However, stent occlusion is a known complication, necessitating close monitoring and timely removal once the acute episode resolves. Early removal of the biliary stent was essential in this case to prevent additional complications and optimize patient outcomes.

Routine ultrasound screening in high-risk pregnancies could play a pivotal role in preventing complications. In this case, earlier monitoring might have identified the gallstones at a stage when additional preventive measures could have been implemented. Incorporating gallstone screening into prenatal care protocols, especially for women receiving prolonged progesterone therapy, could improve maternal outcomes [12]. The size of the gallstones plays a crucial role in determining the need for surgical intervention. Stones smaller than 5 mm are more likely to pass spontaneously through the bile ducts without causing obstruction, while larger stones often lead to complications such as jaundice or pancreatitis. Non-surgical options, such as UDCA therapy, are particularly effective for smaller, cholesterol-based stones, especially in patients who have contraindications to surgery.

Pharmacological interventions remain a cornerstone of conservative gallstone management. The use of UDCA to dissolve cholesterol stones and omega-3 supplementation to improve bile composition demonstrated effectiveness in this case. These therapies, along with dietary modifications, offer noninvasive solutions to managing gallstones in pregnancy and postpartum [13].

Conservative management typically involves pain relief, hydration, and dietary interventions. Studies suggest that many pregnant women with uncomplicated gallstone disease can successfully avoid surgical intervention until after delivery, minimizing maternal and fetal risks. At the same time, conservative treatment, while effective in symptom management, carries risks of recurrence and progression to more severe complications such as cholecystitis or pancreatitis. Relapse rates are particularly high during pregnancy, necessitating a proactive approach to monitoring and intervention [14].

Developing standardized protocols for the conservative management of gallstones during pregnancy is essential. Future studies should focus on long-term maternal and fetal outcomes, the role of pharmacological agents like UDCA, and identifying predictors for the need for surgical intervention. However, complications such as bile duct obstruction may necessitate endoscopic or surgical intervention. Laparoscopic cholecystectomy remains the gold standard for symptomatic gallstones during pregnancy, particularly in the second trimester. Recent studies indicate that laparoscopic surgery can be safely performed in all trimesters with minimal risk to both the mother and fetus. In cases where bile duct obstruction occurs, surgical intervention may become necessary to prevent further complications [15].

General anesthesia and major surgical procedures performed in the immediate postpartum period can significantly affect breastfeeding initiation and maternal-infant bonding. Anesthesia medications, such as propofol and opioids, can pass into breast milk, potentially impacting the newborn. However, most guidelines suggest that brief interruptions in breastfeeding (typically 24-48 hours) are safe [16]. Research indicates that breastfeeding can usually resume without long-term effects when properly managed. Nonetheless, the stress of surgery and recovery may delay the establishment of breastfeeding, with some studies suggesting a higher risk of early breastfeeding cessation among postpartum surgical patients [17].

Future research should focus on the long-term benefits of combining omega-3 fatty acids with UDCA to prevent gallstone formation during pregnancy. Additionally, exploring the effects of elevated progesterone levels on gallbladder motility and investigating pharmacological interventions to counteract these effects could offer new insights into treatment strategies. Further studies are needed to evaluate the efficacy of routine ultrasound screening in reducing gallstone-related complications. Such studies would be valuable in refining clinical management practices and improving patient outcomes during pregnancy.

## Conclusions

This study demonstrates the success of a multidisciplinary, conservative approach to managing pregnancy-associated gallstone disease. Using UDCA and omega-3 fatty acids (DHA and EPA) dissolved cholesterol gallstones postpartum, while ERCP resolved acute biliary obstruction. The patient-centered approach prioritized maternal bonding and breastfeeding, leading to excellent maternal and neonatal outcomes without immediate surgery.

The case highlights the value of routine ultrasound screening in high-risk pregnancies for early detection and management. It also calls for further research into the long-term benefits of UDCA, omega-3 supplementation, and methods to address progesterone-induced gallbladder stasis. Advancing non-surgical therapies and individualized care can reduce risks and improve outcomes for pregnant women with gallstone disease.

## References

[1] Ko CW, Beresford SA, Schulte SJ, Matsumoto AM, Lee SP (2005). Incidence, natural history, and risk factors for biliary sludge and stones during pregnancy. Hepatology.

[2] de Bari O, Wang TY, Liu M, Paik CN, Portincasa P, Wang DQ (2014). Cholesterol cholelithiasis in pregnant women: pathogenesis, prevention and treatment. Ann Hepatol.

[3] Méndez-Sánchez N, González V, Aguayo P, Sánchez JM, Tanimoto MA, Elizondo J, Uribe M (2001). Fish oil (n-3) polyunsaturated fatty acids beneficially affect biliary cholesterol nucleation time in obese women losing weight. J Nutr.

[4] Jang SI, Fang S, Kim KP (2019). Combination treatment with n-3 polyunsaturated fatty acids and ursodeoxycholic acid dissolves cholesterol gallstones in mice. Sci Rep.

[5] Weiner E, Mizrachi Y, Keidar R, Kerner R, Golan A, Sagiv R (2015). Laparoscopic surgery performed in advanced pregnancy compared to early pregnancy. Arch Gynecol Obstet.

[6] Pearl JP, Price RR, Tonkin AE, Richardson WS, Stefanidis D (2017). SAGES guidelines for the use of laparoscopy during pregnancy. Surg Endosc.

[7] Hedström J (2023). Management of Gallstone Disease in Pregnancy. Aspects on Intervention, Outcome and Patient Experience. https://portal.research.lu.se/en/publications/management-of-gallstone-disease-in-pregnancy-aspects-on-intervent.

[8] Mendez-Sanchez N (2006). Pregnancy and gallbladder disease. Ann Hepatol.

[9] Saddique MN, Saleem S, Shahid I (2024). The estrogen-gallstone connection: uncovering the pathways. Discov Public Health.

[10] Gilat T, Konikoff F (2000). Pregnancy and the biliary tract. Can J Gastroenterol.

[11] Berr F, Holl J, Jüngst D, Fischer S, Richter WO, Seifferth B, Paumgartner G (1992). Dietary N-3 polyunsaturated fatty acids decrease biliary cholesterol saturation in gallstone disease. Hepatology.

[12] European Association for the Study of the Liver (EASL) (2016). EASL Clinical Practice Guidelines on the prevention, diagnosis and treatment of gallstones. J Hepatol.

[13] Cho SM, Park JA, Kim NH (2015). Effect of eicosapentaenoic acid on cholesterol gallstone formation in C57BL/6J mice. Mol Med Rep.

[14] Ghumman E, Barry M, Grace PA (1997). Management of gallstones in pregnancy. Br J Surg.

[15] Nan X, Chan E, Wong KS, Ng J, Izwan S, Cooper M, Damodaran R (2023). Laparoscopic cholecystectomy in pregnancy: a seven-year retrospective study from an Australian tertiary center. Cureus.

[16] Reece-Stremtan S, Campos M, Kokajko L (2017). ABM clinical protocol #15: analgesia and anesthesia for the breastfeeding mother, revised 2017. Breastfeed Med.

[17] Pop R, Cebula H, Lambert A (2020). Treatment with flow diverter stent during pregnancy. Neuroradiology.

